# What, who, and when? How social networking achieves online digital engagement in an architectural design studio

**DOI:** 10.1186/s44147-022-00101-8

**Published:** 2022-06-29

**Authors:** Ramy Bakir, Sara Alsaadani

**Affiliations:** 1 Architectural Engineering and Environmental Design Department, Arab Academy for Science, Technology and Maritime Transport, Cairo Campus, Elhorria Moshir Ismail St., Heliopolis, P.O. Box 2033, Cairo, Egypt; 2 Architectural Engineering and Environmental Design Department, Arab Academy for Science, Technology and Maritime Transport, Smart Village Campus, P.O.Box 12577, Giza, B2401 Egypt

**Keywords:** Architectural design studio, Social networking platforms, Online digital engagement, Hybrid design education, Inferential statistics

## Abstract

Engagement is integral to design education; thus, the purpose of this work is to examine how engagement can be maintained digitally, using prevalent social networking tools, to support architectural education in the post-pandemic narrative. This study utilizes a quantitative methodology using inferential statistics to analyze observable online digital engagement on a social networking platform, used as part of a hybrid architectural design studio. The platform’s purpose was to overcome time and distance limitations impinged onto traditional studio set-ups. This research aimed at deciphering the intricacies of “how” digital engagement manifested on the platform, by analyzing engagement from the “what,” “who,” and “when” dimensions, throughout a 16-week semester. Digital engagement manifested in two forms: pro-active and re-active. Pro-actively creating posts evoking emotions or soliciting responses was likely to garner re-active engagement. Attachment inclusion was likely to yield engagement in the form of likes, particularly when the attachment was an image. Increased instructor involvement was associated with greater digital engagement. Findings also illustrate how the platform extended the studio’s temporal boundaries, while aligning with its design activities. Results underline best practices to be adopted in design studio teaching as architecture school transition to online and/or hybrid design education, while acclimatizing to realities of the post-pandemic era. The proposition of a 12-point classification for analyses of interactions occurring on social networking platforms in architectural contexts also adds value, as it may be adopted in future research.

## Introduction

Extensive diffusion of social networking platforms amongst today’s student populations has driven researchers to question their suitability to support tertiary education. Easy access, flexibility, functionality, and communicative properties are some of the reasons cited in the literature behind the pervasive use of social networking (SN) in university settings [4, 57]. An association is found between students’ frequent use of SN platforms and positive perceptions of using SN for educational purposes [35]. A qualitative study seeking to assess the impacts of SN on students’ learning experience [24] indicated that SN use promoted greater interaction with other students, improved students’ mastery of course content, increased opportunities for peer learning, promoted critical thinking, and encouraged students towards self-learning. Moreover, students were also better able to monitor their own learning progress through SN use [24]. Studies also suggest that the use of SN in educational settings may improve their collaborative skills [61], enhance students’ motivation and overall learning experience [8, 10, 26], and improve students’ interaction in the classroom [10]. It is further suggested that universities seeking to produce graduates prepared for international career experiences may encourage students’ SN use, to build on experiences of intercultural competence and cross-cultural communication [18].

Teaching design however has its own specificities. Several studies have highlighted how the characteristics of the pedagogical design studio are different when compared to the typical classroom [3, 5, 13]. Several scholars have highlighted how the design studio has its unique environment with specific potentials and limitations [1, 49, 56]. This is manifested in how within the physical space of the design studio architecture students engage with instructors and fellow students in reflective conversations [55] and develop tacit knowledge, design skills, and values through problem-based learning [30, 50, 55]. In addition to its pedagogical significance, the studio also functions as a social space allowing peer-to-peer discussions, developing a community of practice [36, 37], and conveying the professional architectural culture [11], all of which require higher levels of engagement.

Such engagement is integral to architectural design studio pedagogy and is one of the five essential values leading to a healthy studio [30]. While design teaching/learning includes multiple cognitive styles, there is a consensus that design education requires students to consistently work collaboratively [49], as students profit from debates during collaborative discussions [52].

However, the traditional studio still faces many challenges. Güler [20] acknowledges that studio hours are not sufficient for lengthy discussions. Budget cuts pressure institutions to increase student-to-instructor ratios, reducing time dedicated to each student [41, 53]. Ever-increasing numbers of student enrolment also make traditional “live-in” studios unsustainable [41]. Nevertheless, “for many decades questioning the realities of architectural education and design studio has been a taboo” [51]. It is the recent onset of the coronavirus that further exposed vulnerabilities of architectural education. The tradition’s lack of resilience has been highlighted by sudden disruptions impinged onto design studio practices as schools worldwide accommodate lockdown requirements. This is forcing us to re-question prevailing and inherited practices of architectural education.

The nineteenth century Beaux-Arts and subsequent twentieth Century German Bauhaus atelier studio models continue to have enduring influences on architecture schools today [49]. However, architectural education will need to acclimatize to the challenges of the twentyfirst century, particularly the COVID-19 reality. Many schools have already shifted towards online and/or hybrid learning environments to maintain safe, socially distant yet engaging design studio cultures. This has been enabled via intensive reliance on digital technologies and telecommunications, already common features of daily life at this time.

Students’ experiences and their engagement within the studio have been improved using technology-based learning [25] which creates an augmented reality, changing how students perceive designed artifacts, and accordingly how these artifacts are imagined and represented. Computer technologies also play an important role in shaping what students perceive as more successful paths to design solutions [59]. Rook and Hooper [48] also argue that their potential lies in their ability to enhance design thinking by allowing students to take greater command of developing their own tacit knowledge.

A body of literature on architectural education in the post-pandemic era is currently a work-in-progress; this article seeks to contribute to the emergent discussion. However, several studies already focus on online tools available to maintain engagement with students remotely. In the upcoming section, the potentials of SN, an intrinsic element of students’ daily lives, are reviewed as a means of enhancing higher education. For this research, we use the same definition of SN as that proposed in Lim and Richardson [35], whereby SN platforms are identified as virtual spaces that allow users to do the following:

- Create a profile within an enclosed system that can be viewed either publicly or semi-publicly
- Construct a similar registry of users with whom mutual connections are shared
- View the profiles of those mutual connections and beyond.

There is further evidence of the potential of SN tools to enhance levels of engagement in the pedagogical design process [41, 42], which is “not primarily an instructional process, but rather a process of interaction and experience” [31], especially in the field of architecture. This is why this paper assumes that looking specifically at the context of the design studio within architectural education could shed more light on how SN could be utilized in enhancing engagement in higher education. We subsequently present results of recent empirical work in support of this argument, discussing the intricacies of how SN platforms have garnered digital engagement to support an architectural design studio. This work is concluded by highlighting best practices that could help in maintaining online digital engagement in the studio as we adjust to the novel post-pandemic reality.

## Review of social networking in the architectural design studio

Attention has been shifting toward the internet to overcome afore-stated challenges. For example, virtual design studios (VDS), “a type of studio that … expand [s] studio space beyond physical and time limits” [47], are now in use worldwide (e.g., [22, 23, 47]). Information and communication technologies (ICT) and formal, academic Learning Management Systems (LMS) (e.g., Blackboard, Moodle, etc.), are often used to facilitate VDS setups (e.g., Lotz et al., [37]). This complements conventional course delivery, providing access to course content, and creating a platform for online collaboration and assessment of student works [31].

It is argued however that virtual environments align with instructional teaching approaches and may lead to teaching in-silos, making “distance learning” unpopular [31]. Nevertheless, “teaching architecture is not primarily an iinstructionalprocess, but rather a process of interaction and experience” [31]. Thus, one way of engaging students and enhancing their learning is by evolving to match the “nomadic device generation’s” thinking and learning styles [54].

Schnabel and Ham [54] state that “the next logical step in developing the VDS was collaboration within a [social network].” Recognizing that students nowadays tend to have more advanced technological skills than their instructors, they combined SN with LMS to create a Social Network Learning Cloud (SNLC), to flatten the traditional teacher-student hierarchy, and create a more democratic, team-oriented environment [54].

Increasingly, VDS is being supported by SN platforms (e.g., Pektaş [47]; Schnabel and Ham [54]). Pektaş and Gürel [46] use LMS for interaction and a digital repository of course content but add the SN platform Facebook for asynchronous digital communication. Peer-to-peer collaborations are seen to be enhanced when supported by such platforms [9], especially that smartphone applications have expanded the use of SN in educational contexts [14].

Papacharissi [45] contends that “the architecture of [SNs’] virtual spaces … much like the architecture of physical spaces, simultaneously suggests and enables particular modes of interaction.” SN is therefore becoming the focus of academic research on architectural pedagogy. However, our review reveals that out of all SN platforms, Facebook is most commonly used to support architectural design studios (e.g., [20, 21, 41, 42, 60]), prompting its selection as a case study in this work. Enthusiasm for Facebook is built upon its communicative properties; e.g., allowing peer-to-peer discussions and increasing accessibility of learning resources [9, 58].

Most studies using Facebook as an educational support in the architectural studio tend to be qualitative. In Morkel [41, 42], the focus groups were conducted, gathering insights regarding how a closed Facebook group impacted upon their learning experiences. Findings revealed that the group promoted informal dialog and discussion. In Tate and Osborne [60], several Facebook groups were created to increase tutor-student and peer-to-peer engagement. By drawing on previous studies, group content was sorted into five categories; “excitement,” “problem,” “solution,” “joke,” and “other.” Results indicated that the most successful groups were those in which tutors were actively involved. In McCarthy [38, 39], Facebook was used to set up a virtual classroom and online gallery for students’ design development.

Güler [20] compares the effectiveness of a traditional studio with a design studio conducted on Facebook using a quantitative, experimental procedure. Students were divided into two groups; a control group attended a physical studio and a treatment group experienced an online studio. Student feedback indicated the encouragement of participation and the platform’s continuous interaction capabilities contributed to the success of the online studio.

Our review indicates that SN platforms such as Facebook hold tremendous potential to support the architectural design studio. However, to date, a single comprehensive quantitative study deciphering intricacies of how engagement is achieved when SN tools are used to support design studios ceases to exist. We therefore seek to understand **how users interact and engage on a Facebook group, the SN platform studied in this research,** by answering the following research questions:

- **What** is the nature of the content posted on the platform? What types of posts tend to yield more likes or comments?
- **Who** creates posts? Who responds?
- **When** is the platform most commonly used?

This may allow us to understand how SN platforms foster digital engagement and support hybrid design studios, ensuring that design education achieves its goals even when conducted remotely.

## Online digital engagement

“Engagement” is the psychological state of enjoyment toward an object or action [33]. In higher education, engagement refers to the “quantity and quality of the physical and psychological energy that students invest” [2]. Valerio et al. [62] outline four kinds of student engagement occurring in universities:

- **Academic engagement** with curricular activities.
- **Social engagement** with the university community’s social activities.
- **Brand engagement** with universities as educational service providers.
- **Digital engagement** with digital content shared online.

Studying digital engagement is integral for design studio teaching/learning. There are various means available at the instructor’s disposal to keep students engaged in physical settings [25], but keeping students engaged online is more challenging, which prioritizes the need to explain digital engagement as a step towards enhancing it.

In this study, we focus on observable digital engagement. Accordingly, two assumptions must be met [12]. One is that digital engagement with SN can be observed through:

- Post content.
- Post media type.
- The time the post was made.

These elements are considered independent variables in this study. While previous studies (e.g., [12, 62]) have studied digital engagement in public groups with a broad user base, this contribution focuses on a private Facebook group with a limited number of users. Therefore, in addition to the above elements, we investigate who created the post, which establishes the three dimensions of inquiry; “what,” “who,” and “when.”

The second assumption is that the numbers of likes and comments are measures of engagement [12, 40, 62]. In previous studies, quantifying likes, comments, and shares allowed researchers to confirm the existence of statistically significant relationships between digital engagement and the time a post is created [12], and a post’s media type [12, 40, 62]. Facebook groups investigated in the afore-stated studies are public groups, and since this Facebook group under study is closed, shares are not included as a measure of engagement and only numbers of likes and comments are analyzed as dependent variables.

## Methods

### The studied studio

The studio under scrutiny here is the second design studio taught to the undergraduate architecture students at a non-governmental higher education institution in Cairo, Egypt. This studio comprised 43 students and was led by an instructor who has been teaching this studio since 2013. The studio was conducted in a fall semester prior to the COVID-19 pandemic and was held for eight hours a week (Sundays and Wednesdays), with support from five teaching assistants. Students were required to design one project, throughout the 16-week semester, which was divided into 7 design-stages.

### The studied platform

A closed Facebook group was created 8 semesters prior to the studied fall semester, to increase the contact time between instructors and students, provide course material, and make announcements. The group has been used for the following cohorts since, accumulating 271 members. This allowed for a hybrid studio where communication occurred on campus during studio hours and on the platform otherwise.

At the beginning of the semester, students were asked to join the group and were encouraged to remain active. They all already had Facebook accounts, and all joined within the first week of the semester. Online involvement was not mandatory and was not incentivized. All students were informed that the group would be monitored for this study, and the nature of which was fully disclosed. Throughout the semester, a total of 122 posts were produced by group members, and none of them was altered nor removed.

### Data coding and analysis

At the end of the semester, the studied Facebook group activity was analyzed to identify the following:

1. **Who** created each post, where the study identified for each post “who” posted it:

- Faculty/student
- If by students, whether they were enrolled during the semester under study (i.e., currently enrolled) or previously enrolled.

2. **What** each post consisted the following:

- Post classification. Moore-Russo et al. [40] categorized posts into three broad categories and eleven sub-categories, but the study is unrelated to design studio contexts. In Tate and Osborne’s [60], a study conducted in the architectural design studio, a five-point categorization is used. However, this is originally derived from English and Duncan-Howell’s [15] study belonging to the business education context and was not considered comprehensive enough to describe the breadth of activity occurring on the platform, especially one that it is used specifically for architectural purposes. This is why the authors of this study have developed a 12-point classification to describe “what” kind of posts were made on the studied group, and they consisted of the following categories: *(tutor instructions – tutor announcements – design precedents – presentation precedents – class activity outcomes – student-to-student communication – peer-to-peer learning – tutor student social interaction – student-tutor enquiries – TA assistance – fun – tutorials).*
- Whether the post was “shared.”
- Whether the post contained an attachment.
- Type of attachment included *(none – image – video – link – files – multiple).*

3. **When** each post was created on the studied group:

- In terms of week number.
- And in terms of day of the week.

**Fig. 1:**
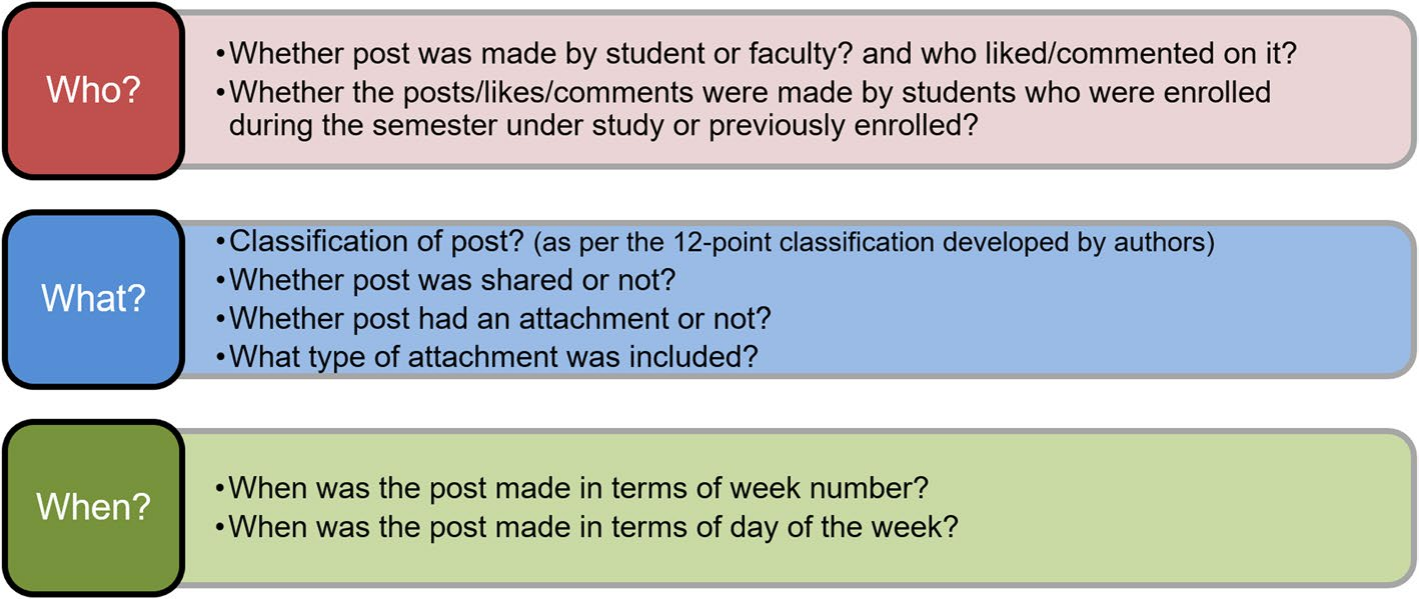
The three main parameters focused on regarding the data collected for each of the 122 posts made on the group during the studied period

The identification of the three parameters of who, what, and when was then followed by the analysis of the likes and comments on each post (Fig. 1), where they were coded to understand how group members engaged, as shown below:

- Total number of likes/comments.
- And again ‘who’ those likes/comments were made by the following:
- Faculty/student
- Currently/previously enrolled students.

**Fig. 2:**
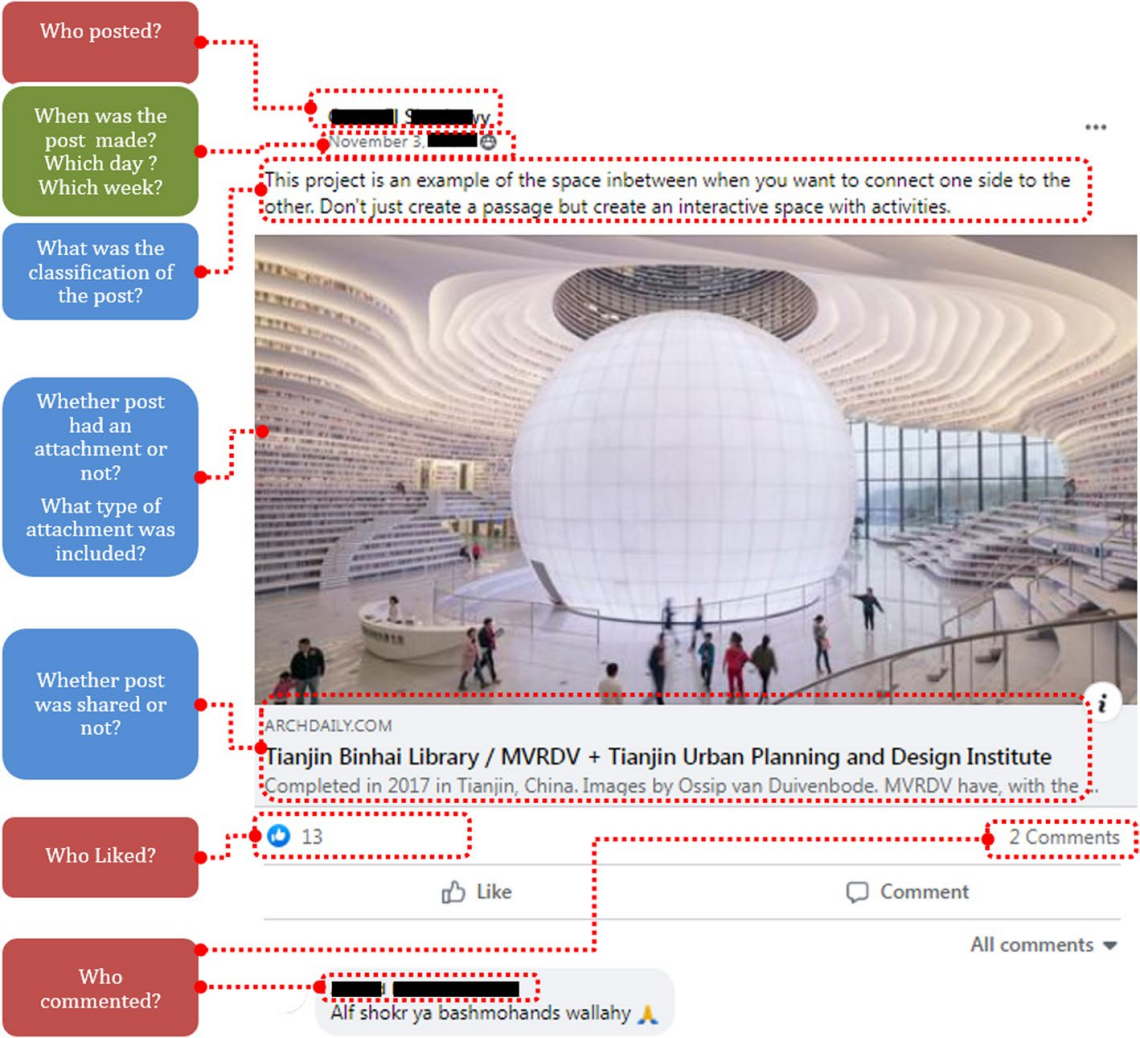
A sample of how the activities on the Facebook group were analyzed according to the three parameters of the study (“Who,” “What,” “When”)

The analyzed activities in the form of posts, likes, and comments that manifested on the group (Fig. 2) were coded onto a Microsoft Excel spreadsheet to then they were imported into the statistical software package SPSS for descriptive and inferential statistical analyses. As data did not meet assumptions of normality, non-parametric tests were used. The power analysis (section Power analysis) was computed using G*Power 3 [16].

## Results

### Preliminary analyses

#### Descriptive statistics

**Fig. 3:**
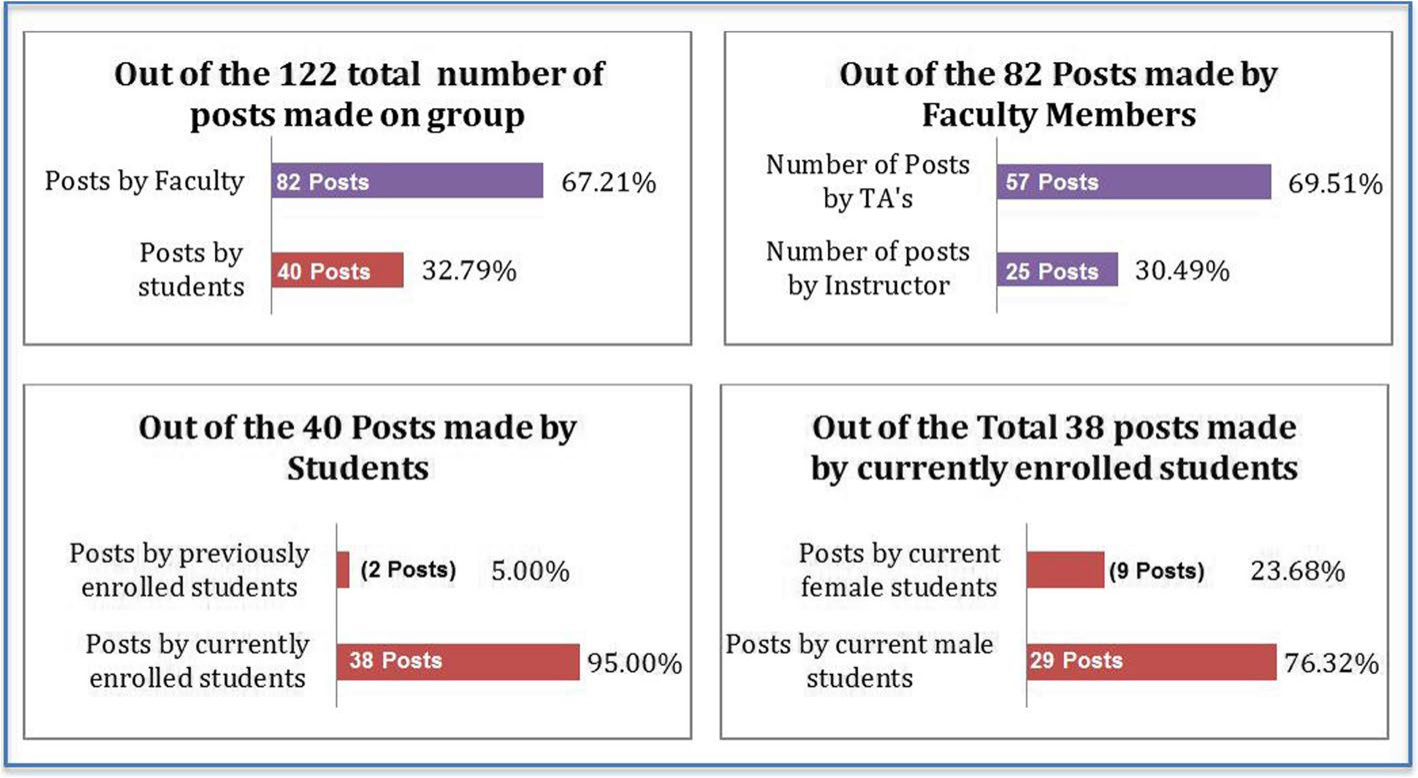
Bar charts presenting numbers and percentages of the posts made on the group in regards of “Who” is posting them

The preliminary data analysis indicated that out of the 122 posts made on the group the students only posted 40 posts, where the faculty posted 82 posts, 25 of which were made by the studio instructor and the rest by the teaching assistants (TA’s) as shown in Fig. 3 below. The majority of the posts made by students were made by students who were registered to the course at the time of the study in the form of 38 posts, 29 of which were made by male students as shown in Fig. 3 below.

**Fig. 4:**
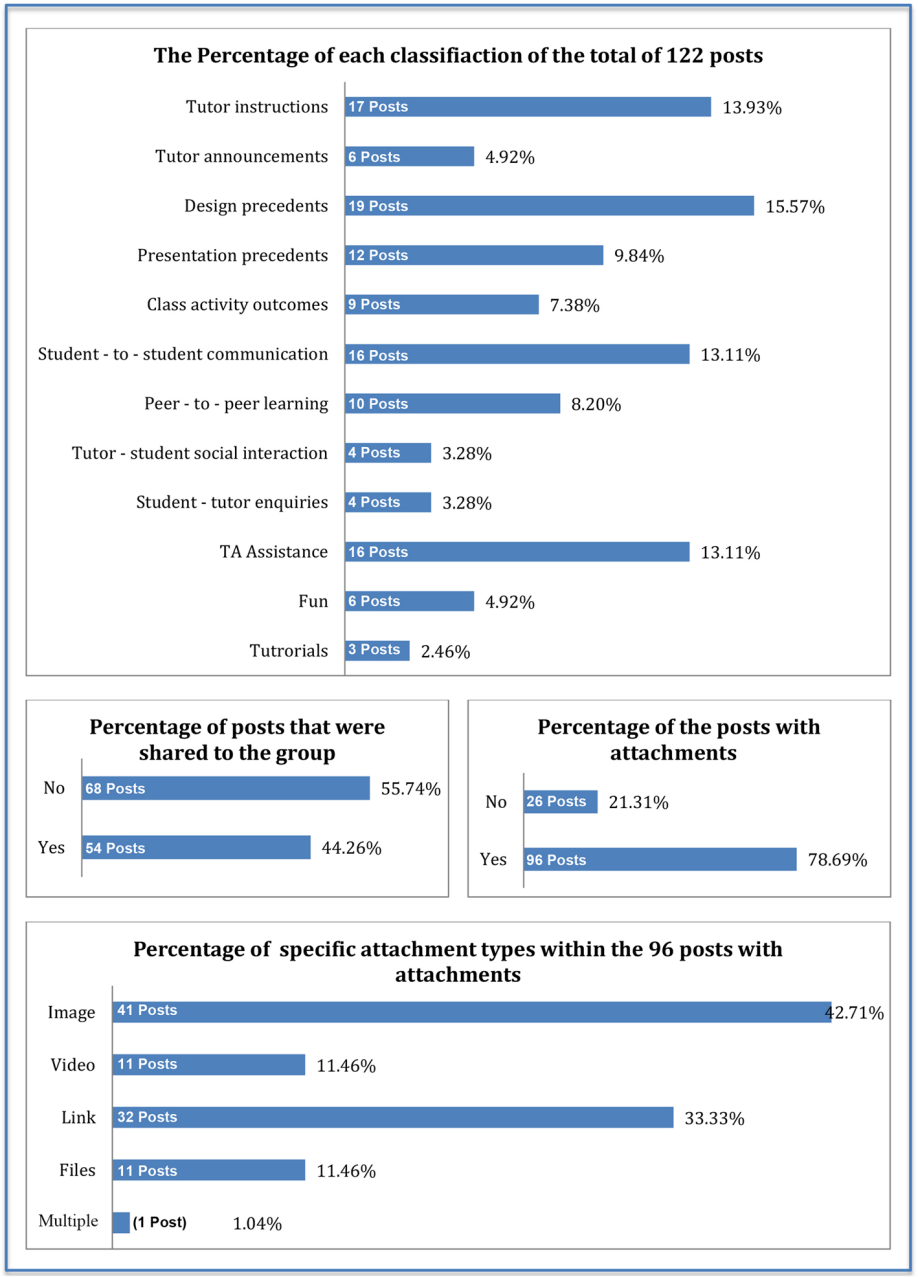
Bar charts presenting the number and percentage of posts made in the group in regards of “What” was posted and “What” was the medium of the post

Descriptive statistics regarding the three parameters of “what,” “who,” and “when” of the posts created on the group as explained in section Data coding and analysis. Regarding thematic classification, the largest post category was “design precedents” (19 posts), followed by 17 “tutor instructions” as shown in Fig. 4 below. Fifty-four posts were shared from other Facebook accounts, and 96 included attachments (Fig. 4).

**Fig. 5:**
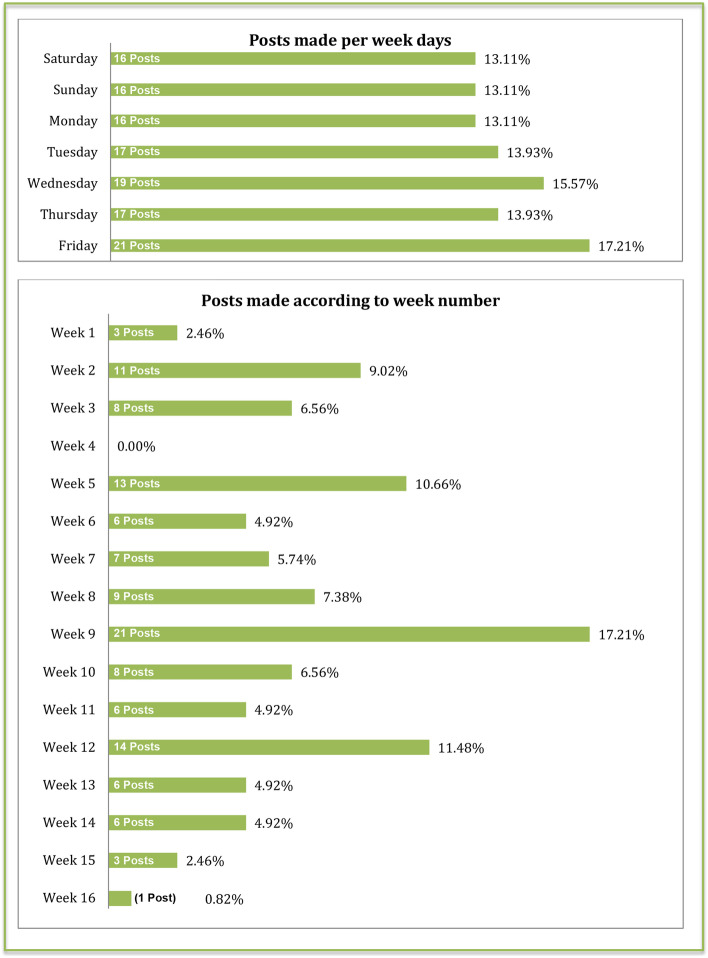
Bar charts presenting the number and percentage of posts made in the group in regards of “When” they were posted

Numbers of posts were uniform during weekdays as shown in Fig. 5 below, with a slight increase on Fridays; the only day of weekend the students took that semester. Also, over 17% of posts were made during week 9, just after the first mid-term submission.

**Fig. 6:**
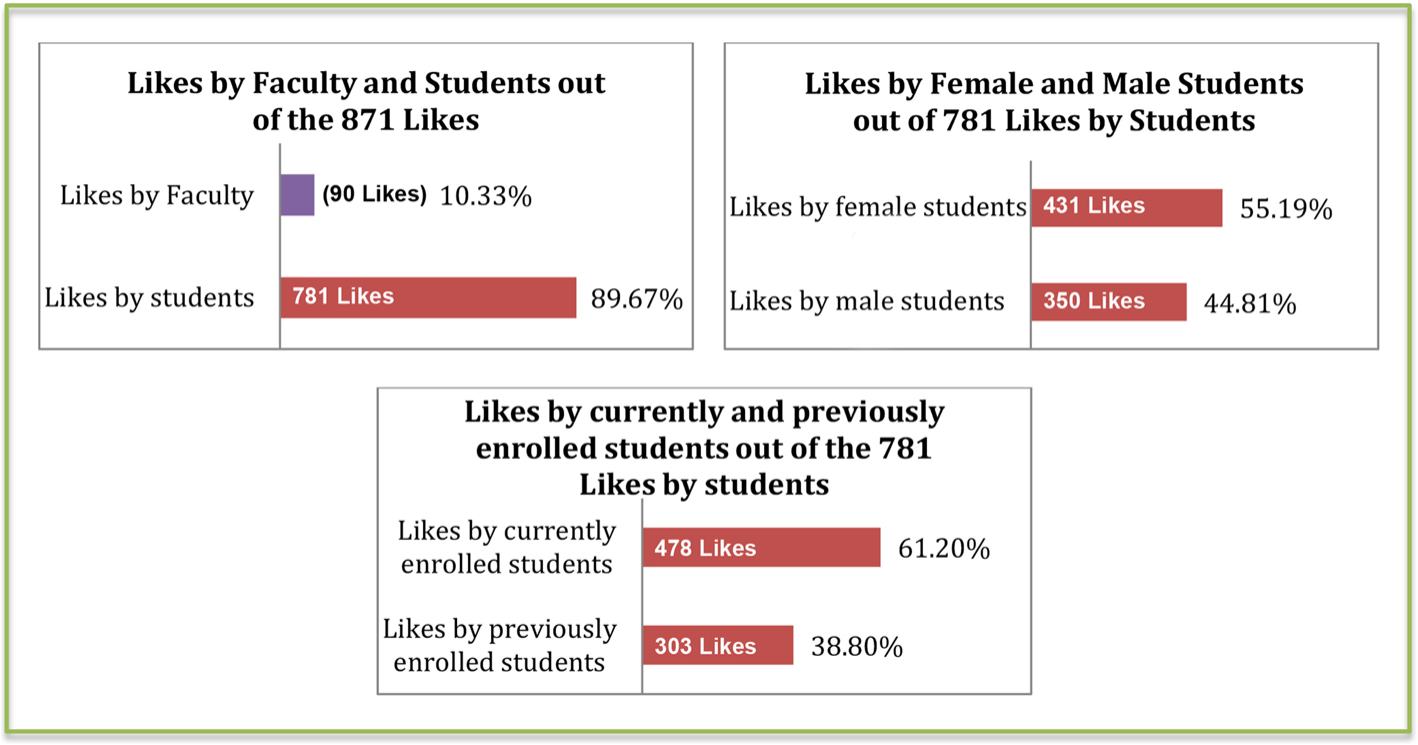
Bar charts presenting the number and percentage of likes made in the group in regards of “Who” made them

**Fig. 7:**
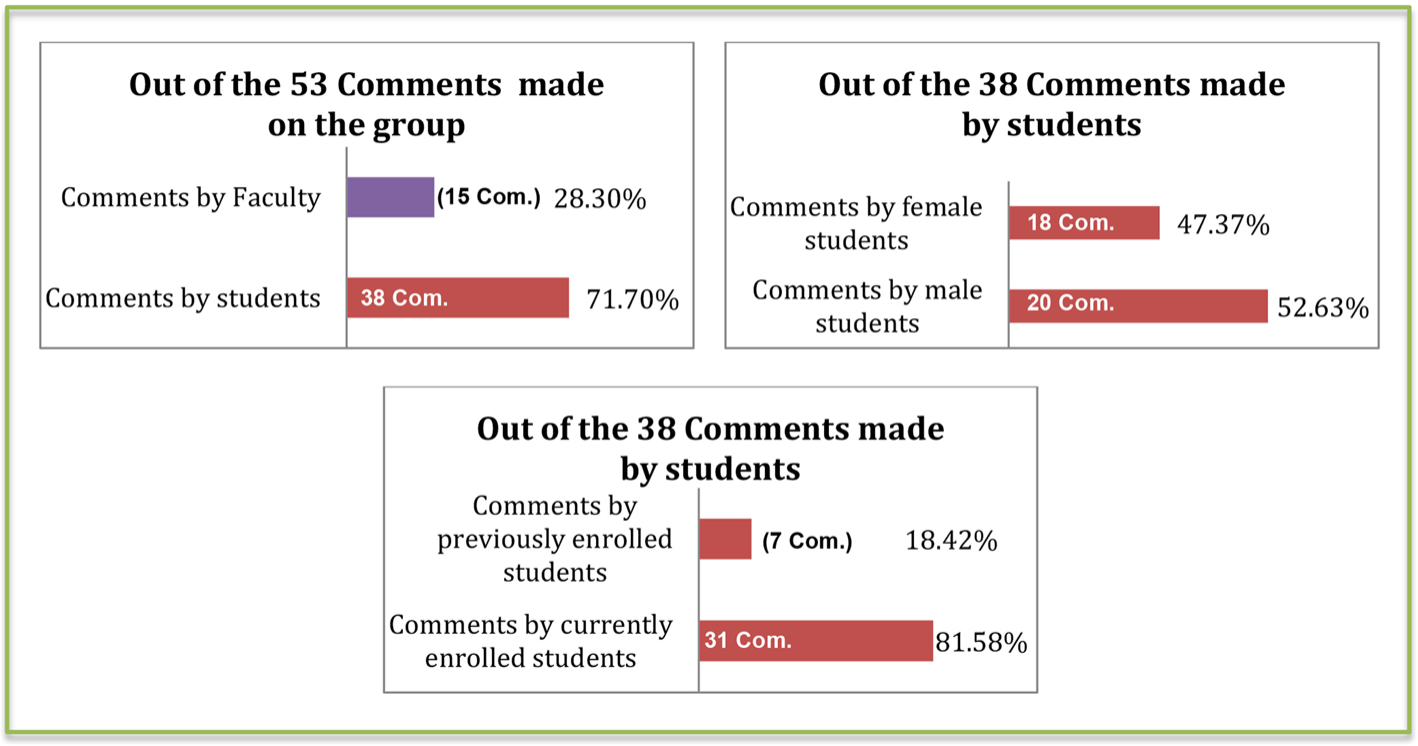
Bar charts presenting the number and percentage of comments made in the group in regards of “Who” made them

In total, 871 likes were made to posts (Fig. 6); 90 by faculty, the rest by students. Only 53 comments were made; 38 were by students (Fig. 7).

#### Power analysis

The coding scheme meant that each post was treated as an individual content unit. Thus, the sample constituted 122 posts; 82 by faculty and 40 by students. A post hoc power analysis was performed to ascertain the statistical power of this sample using the non-commercial software G*Power3 [16], testing the difference between two groups using a one-tailed Mann-Whitney *U* test, a medium effect size *(d=0.50)* and an alpha of *.05.* Results showed that a sample of 122 posts, with unequal sized groups (*group 1, n=82; group 2, n=40)* achieved a power *(P 1 - β)* of *0.81 or 81%*, exceeding the required level of 0.8 (e.g., [34]). This indicates that, despite the moderately-sized sample, results of inferential tests are statistically reliable.

### Inferential statistics

Results of inferential tests are shown and summarized in Table 1, and significant relationships will be presented here along the what, who, and when dimensions.

**Table 1:** Summary of results—table of significances. Significant results are highlighted

Independent variables	Likes by					Comments by				
	All group members (Total)	Faculty	Students	Current students	Previous students	All group members (Total)	Faculty	Students	Current students	Previous students
**‘What’ variables**										
**Post classification**	.000***	.049*	.000***	.000***	.119	.009**	.003**	.023*	.116	.182
**Shared or not?**	.834	.023*	.652	.917	.976	.400	.472	.446	.928	.409
**Attachment or not?**	.007**	.883	.013*	.006**	.052	.141	.172	.158	.223	.046*
**Attachment-type**	.041*	.467	.044*	.038*	.025*	.141	.373	.312	.780	.291
**‘Who’ variables**										
**Faculty or student members**	.001***	.016*	.001***	.021*	.040*	.224	.057	.710	.359	.797
**Currently or previously enrolled students?**	.057	.332	.065	.125	.309	.057	.332	.065	.125	.309
**‘When’ variables**										
**Week number**	.376	.022*	.501	.383	.005**	.003**	.013*	.002**	.000***	.111
**Day of the week**	.135	.193	.142	.501	.159	.284	.297	.212	.325	.648

*Significant at *p* < .05 level

**Significant at *p* < .01 level

***Highly significant at *p* < .001 level

All results are for a 95% confidence interval

#### “What?”

##### Post-classification

A series of Kruskal-Wallis *H* tests revealed consistently significant relationships ^[^1^]^ between the 12-point classification system developed and used by the authors (section Data coding and analysis) and likes and comments (Table 1).

A Kruskal-Wallis *H* test revealed a highly significant relationship between post-classification and total number of likes; *X*^*2*^*(11)=34.16, p=0.000.* The highest mean rank was identified in two of the twelve categories of posts made on the studied group and those were first, the “tutor-student social interaction” category *(119.50)*, followed by the second category of *“*fun” *(82.50)* (Table 2). These categories achieved the highest mean ranks, although there were fewer cases of these amongst the post sample (Fig. 4). In regards to engagement on the SN, similar significances were found between post-classification and likes by the following:

- Faculty *(X*^*2*^*(11)=19.743,p=0.049)*
- Students *(X*^*2*^ *(11)=34.429,p=0.000)*
- Current students *(X*^*2*^ *(11)=36.71,p=0.000).*

**Table 2:** Significant results of Kruskal-Wallis H tests. Only significant results are shown

**Dependent variable**	**‘What?’** ***Independent variable: Post classification (Categorical variable)***						
	Categories	*N*	Mean ranks	sd	*X*^2^	df	*p*
Total number of likes	Tutor instructions	17	48.21	9.05	34.16	11	.000***
	Tutor announcements	6	45.17				
	Design precedents	19	68.66				
	Presentation precedents	12	68.66				
	Class activity outcomes	9	63.33				
	Student-to-student communication	9	50.94				
	Peer-to-peer learning	16	42.30				
	Tutor-student social interaction	10	119.50				
	Student-tutor enquiries	4	11.63				
	TA assistance	4	78.31				
	Fun	6	82.50				
	Tutorials	3	67.00				
Likes by faculty	Tutor instructions	17	69.88	1.61	19.743	11	.049*
	Tutor announcements	6	64.74				
	Design precedents	19	61.63				
	Presentation precedents	12	59.04				
	Class activity outcomes	9	71.78				
	Student-to-student communication	9	51.09				
	Peer-to-peer learning	16	47.20				
	Tutor-student social interaction	10	110.38				
	Student-tutor enquiries	4	35.50				
	TA assistance	4	62.97				
	Fun	6	49.50				
	Tutorials	3	74.50				
Likes by students	Tutor instructions	17	50.65	6.31	34.249	11	.000***
	Tutor announcements	6	40.67				
	Design precedents	19	68.42				
	Presentation precedents	12	68.38				
	Class activity outcomes	9	60.50				
	Student-to-student communication	9	50.81				
	Peer-to-peer learning	16	42.35				
	Tutor-student social interaction	10	119.13				
	Student-tutor enquiries	4	12.38				
	TA assistance	4	79.34				
	Fun	6	83.75				
	Tutorials	3	66.17				
**Dependent variable**	**‘When; during the semester?’** ***Independent variable: Week number (Categorical variable)***						
	Categories	N	Mean ranks	sd	X^2^	df	*p*
Likes by faculty	Weeks 1-3	22	79.52	1.61	14.764	6	.022*
	Weeks 4-6	19	63.74				
	Week 7	7	35.50				
	Weeks 8-11	44	56.63				
	Week 12	14	67.18				
	Weeks 13-15	15	55.37				
	Week 16	1	35.50				
Likes by previous students	Weeks 1-3	22	71.95	4.50	18.629	6	.005**
	Weeks 4-6	19	72.64				
	Week 7	7	61.64				
	Weeks 8-11	44	64.88				
	Week 12	14	44.71				
	Weeks 13-15	15	39.00				
	Week 16	1	39.00				
Total number of comments	Weeks 1-3	22	60.50	.97	20.194	6	.003**
	Weeks 4-6	19	74.97				
	Week 7	7	91.50				
	Weeks 8-11	44	52.85				
	Week 12	14	56.46				
	Weeks 13-15	15	62.90				
	Week 16	1	47.50				
Comments by faculty	Weeks 1-3	22	58.23	.37	16.113	6	.013*
	Weeks 4-6	19	71.92				
	Week 7	7	72.64				
	Weeks 8-11	44	55.50				
	Week 12	14	59.79				
	Weeks 13-15	15	67.50				
	Week 16	1	55.50				
Comments by students	Weeks 1-3	22	59.75	.74	21.003	6	.002***
	Weeks 4-6	19	76.08				
	Week 7	7	90.86				
	Weeks 8-11	44	54.47				
	Week 12	14	58.11				
	Weeks 13-15	15	56.53				
	Week 16	1	49.00				
Comments by current students	Weeks 1-3	22	52.00	.61	28.946	6	.000***
	Weeks 4-6	19	74.79				
	Week 7	7	94.57				
	Weeks 8-11	44	60.06				
	Week 12	14	60.25				
	Weeks 13-15	15	56.00				
	Week 16	1	56.00				

*Significant at *p* < .05 level

**Significant at *p* < .01 level

***Highly significant at *p* < .001 level

All results are for a 95% confidence interval

For all these results, the highest mean rank was in “tutor-student social interaction” (Table 2).

As for the of engagement with the content on the SN in the form of comments, a Kruskal-Wallis *H* test also revealed a significant relationship between post-classification and total number of comments; *X*^*2*^*(11)=24.901, p=0.009.* The highest mean rank was for “student-tutor enquiries” *(94.25),* followed by “tutor announcements” *(86.00)* (Table 2). A similar significant relationship was found between post-classification and number of comments by faculty; *X*^*2*^*(11)=28.247, p=0.003,* with a higher mean rank for “student-tutor enquiries” *(100.50)*; indicating that faculty members commented on *“student-tutor enquiries”* more than other categories. A significant relationship was found between comments by students and post-classification; *X*^*2*^*(11)=22.187, p=0.023*, with “tutor announcements” attaining the highest mean rank *(88.42)*; meaning that students are more likely to comment on tutor announcements than other post-categories.

##### Shared posts

A Pearson’s chi-square revealed a highly significant relationship between post-classification and shared posts, *X*^*2*^*(11, N=122)=64.699, p=.000*, indicating which category of posts utilized shared content*.* “Design precedents,” “presentation precedents,” and “peer-to-peer learning” contained higher numbers of shared posts than original posts. Mann-Whitney *U* tests were used to explore whether original posts tend to yield more likes than shared posts. A significant result was yielded for likes by faculty *(U =1440.5, p =.023)* which indicated that the impact of original content was the most on faculty members, who were more likely to like original posts *(mean rank =67.32)* than shared posts *(mean rank =54.18).* No significance was found between comments and shared posts.

##### Attachment

A Pearson’s chi-square between post-classification and posts including attachments yielded a highly significant result, *X*^*2*^*(11, N=122)=72.861, p=.000.* A Pearson’s chi-square between post-classification and attachment-type also yielded a highly significant result; *X*^*2*^*(55, N=122)=175.498, p=.000.* A cross-tabulation between post-classification and attachment-type revealed the following:

- Attachments to “presentation precedents” and “class activity outcomes” were either web-links or images.
- Images were common in “peer-to-peer learning,” “fun,” and “TA assistance.”
- *“*TA assistance” and “design precedents” posts included web-links.
- Attachments to “student-to-student communication” posts mainly included (CAD) files that students circulated.

Mann-Whitney *U* tests were undertaken to ascertain whether posts including attachments tend to yield likes. A significant relationship was confirmed *(U=955, p=.007, attachment included mean rank=66.51, no attachment mean rank=46.81).* Similarly, significant results were yielded between attachment inclusion and likes by the following:

- Students *(U=990.500, p=.013)*
- Current students *(U=948.00, p=.006)* (Table 3).

**Table 3:** Significant results of Mann-Whitney U tests. Only significant results are shown

**Dependent variable**	**‘What?’** ***Independent variable: Attachment or not? (Categorical variable).***					
		*N*	Mean ranks	sd	U-statistic	*p*
Total number of likes	Attachment included	91	66.51	9.05	955.00	.007**
	No attachment	31	46.81			
Likes by students	Attachment included	91	66.12	8.48	990.50	.013*
	No attachment	31	47.95	3.70	959.00	.007**
Comments by previous students	Attachment included	91	59.99	.28	1273.50	.046*
	No attachment	31	65.92			
**Dependent variable**	**‘Who?’** ***Independent variable: Faculty or student member? (Categorical variable).***					
	**Categories**	N	Mean ranks	sd	U-statistic	*p*
Total number of likes	Posts by faculty member	82	68.93	9.05	1030.50	.001***
	Posts by student member	40	46.23			
Likes by faculty	Posts by faculty member	82	66.32	1.61	1244.50	.016*
	Posts by student member	40	51.61			
Likes by students	Posts by faculty member	82	69.09	8.48	1017.5	.001***
	Posts by student member	40	45.94			
Likes by current students	Posts by faculty member	82	55.69	4.92	874	.040*
	Posts by student member	40	43.81			

*Significant at *p* < .05 level

**Significant at *p* < .01 level

***Highly significant at *p* < .001 level

All results are for a 95% confidence interval

However, attachment inclusion does not have a significant impact on number of comments, with exception of comments by previous students. A Kruskal-Wallis *H* test further revealed a significant relationship between attachment-type and total number of likes, *X*^*2*^*(5)=11.576, p=.041*, with the highest mean rank for *image (74.95)*, followed by *link (mean rank=62.03)* (Table 2). Similar significances were found between attachment-type and likes by the following:

- Students (*X*^*2*^*(5)=11.408, p=.044)*
- Current students (*X*^*2*^*(5)=11.777, p=.038)*
- Previous students (*X*^*2*^*(5)=12.865, p=.025).*

Images consistently attained the highest mean rank (Table 2). However, attachment-type is not significantly related to number of comments, based on results of Kruskal-Wallis *H* tests.

#### “Who?”

Pearson’s chi-square tests were performed to investigate whether significant relationships exist between post-creator and post-classification and to be able to identify specific correlations between “who” posted “what.” The only significant relationship was between the “faculty or student” variable from one side, and post-classification from the other, *X*^*2*^*(11, N=122)=108.134, p=000*, which indicated who posted which categories of posts the most, and that was as follows:

Categories of posts exclusively made by faculty were as follows:

- *Tutor instructions*
- *Tutor announcements*
- *Design precedents*
- *Presentation precedents*
- *Tutor-student interaction*
- *TA assistance*

Categories of posts exclusively made by students were as follows:

- *Student-to-student communication*
- *Peer-to-peer learning*
- *Student-tutor enquiries*

Mann-Whitney *U* tests identified significant relationships between post-creator and numbers of likes and/or comments. A highly significant relationship was found between post-creator and total number of likes *(U=1030.5, p =.001).* Faculty posts attained a higher mean rank *(68.93)* than student posts *(46.26).* Similar results were yielded between post-creator and likes by the following:

- Faculty members *(U=1244.5, p=.016)*
- Students *(U=1017.5, p=.001)*
- Current students *(U=1220.5, p=.021)*
- Previous students *(U=874, p=.040).*

Faculty posts consistently attained a higher mean rank (Table 3). No significant relationships were found between the post-creator and number of comments. Students’ enrollment status made no significant difference on likes or comments.

#### When?

##### During the semester

**Fig. 8:**
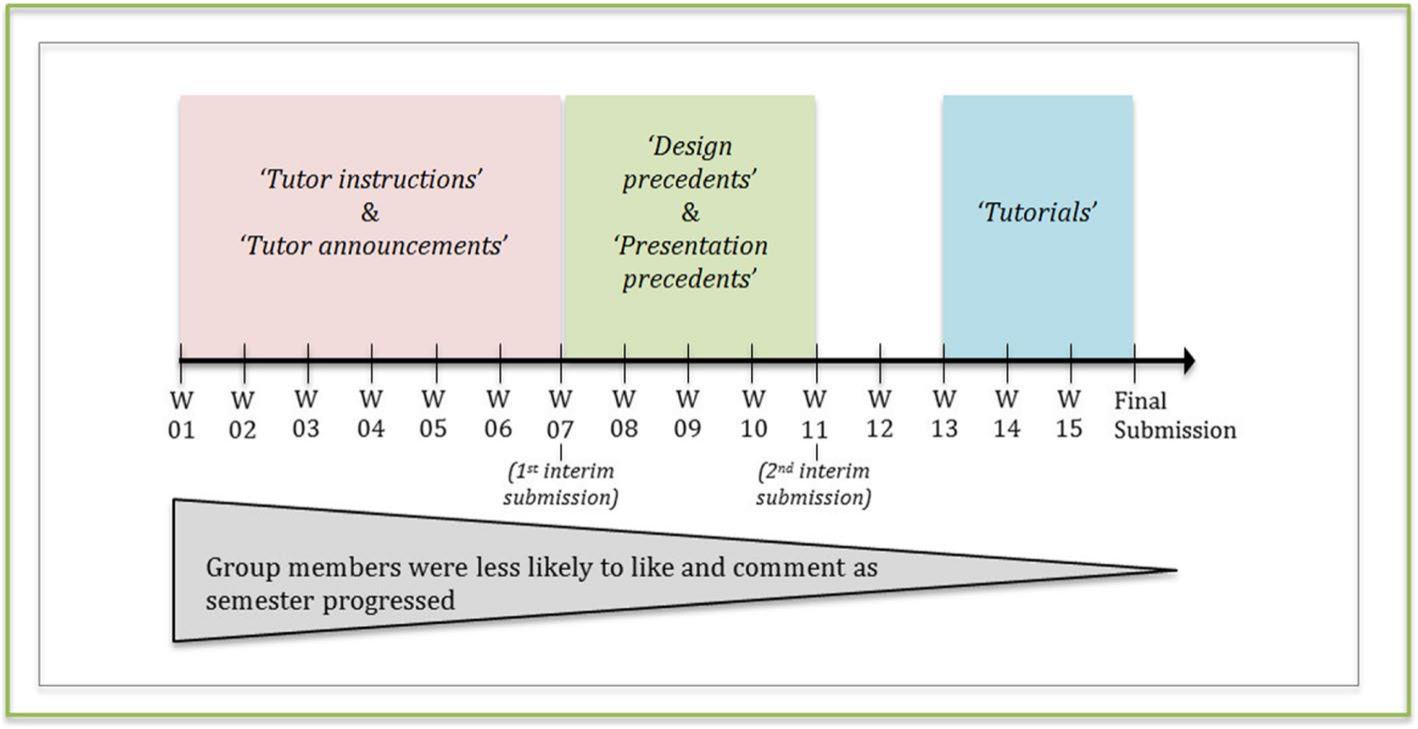
A timeline of the studied academic semester representing the 3 post-classifications of posts most made during the different weeks and their relation to likes and comments made

The relationship between progression of weeks (continuous variable) and post-classification was explored using a Kruskal-Wallis *H* test, but no significant difference was found; *X*^*2*^*(11)=18.048, p=.080.* Nevertheless, a Pearson’s chi-square between week number (categorical) and post-classification yielded a highly significant relationship, *X*^*2*^*(66, N=122)=114.8, p=.000.* A cross-tabulation showed that “tutor instructions” and “tutor announcements” were mostly posted earlier during the semester (weeks 1–3 and 4–6), while “design precedents” and “presentation precedents” were mostly posted halfway (weeks 8–11) as shown in Fig. 8 below. “Tutorials” were only posted prior to final submissions (weeks 13–15), likely to enhance students’ skillsets to complete their work (Fig. 8).

Spearman’s rank order correlations were performed between progression of weeks and numbers of likes and comments. A significant, weak negative correlation was found between progression of weeks and total number of likes (*r*_*s*_*=−.191, p=.018*), likes by faculty *(r*_*s*_*=−.179, p=.024)* and likes by students *(r*_*s*_*=−.158, p=.041).* Similar significant correlations were found between progression of weeks and comments:

- By students (*r*_*s*_*= −.198, p=.028)*
- By previous students *(r*_*s*_*=−.276, p=.001)*
- Total number of comments (*r*_*s*_*=-.159, p =.041)*

This indicates that, in general, as the semester progress, group members are less likely to engage, as they become increasingly busy.

Kruskal-Wallis *H* tests were undertaken to determine whether posts made at a particular point during the semester are more likely to yield likes and/or comments. Significant relationships were found between progression of weeks and:

- Likes by faculty *X*^*2*^*(6)=14.764, p=.022*
- Likes by previous students *X*^*2*^*(6)=18.629, p=.005*
- Total number of comments *X*^*2*^*(6)=20.194, p=.003*
- Comments by faculty members *X*^*2*^*(6)=16.113, p=.013*
- Comments by students *X*^*2*^*(6)=21.003, p=.002*
- Comments by current students *X*^*2*^*(6)=28.946, p=.000.*

The highest mean rank was found between weeks 1–3 for likes by faculty, and between weeks 4–6 for likes by previous students. Conversely, most comments were made during week 7 prior to students’ first interim submission (Table 2). This is with the exception of the result for comments by faculty members, as the mean rank is similar between weeks 4–6 (*mean rank=71.92*) and week 7 *(mean rank=72.64)*; prior to an interim submission during week 8.

##### During the week

A Pearson’s chi-square between post-classification and day of the week a post is created revealed a significant relationship; *X*^*2*^*(66, N=122)=89.109, p=.031.* A cross-tabulation between the two variables, showed the following:

- Most “tutor instructions” were concentrated between the beginning to middle of the week (Saturday-Tuesday).
- “*Tutor announcements”* were made later on the day of the studio to repeat announcements made earlier in-studio.
- *“Design precedents”* were mostly posted after the Wednesday studio.
- *“Presentation precedents”* were posted almost daily.
- *“TA assistance”* posts were made mainly on Saturdays before the Sunday studio.
- *“Student-to-student communication”* and *“peer-to-peer learning”* posts were made between Mondays and Wednesdays.

Kruskal-Wallis *H* tests undertaken to ascertain whether a relationship exists between day of the week a post is made and numbers of likes and/or comments did not yield significant results.

## Discussion

Unraveling “what,” “who,” and “when” dimensions allow us to comprehensively interpret the results and describe “how” patterns of digital engagement manifested, by understanding activities occurring both using, and in response to the 12 post-categories identified by the authors (section Data coding and analysis). Observable digital engagement occurred through post-creation and through likes and comments on posts. This study therefore recognizes that digital engagement manifests in mainly two forms. Posts created represent initiating **pro-active engagement**, while likes and comments represent **re-active engagement** responding to the initial post. The differences between how faculty and students used these forms is discussed throughout the forthcoming section.

### Pro-active engagement

**Fig. 9:**
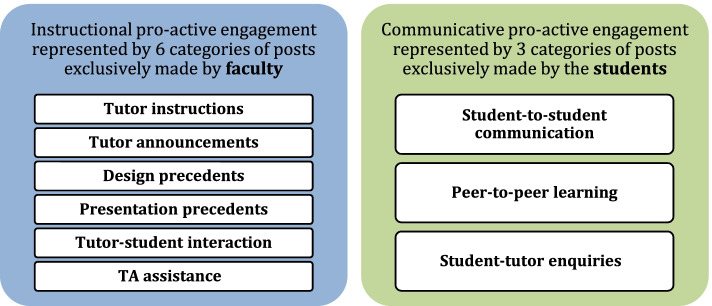
Identified categories of posts as form of pro-active engagement and who posted them

Six post-categories were made exclusively by faculty (section “Who?”). Although these were mainly instructional in nature, they appeared to prompt further interaction on the group, as illustrated in results for “design precedents,” “tutor instructions,” and “tutor announcements” categories, and shown in Fig. 9 below. For instance, “design precedents” was the most commonly posted faculty-created category, was most likely to be posted during the developmental stage of the design process (section During the semester), and was most likely to be made after the week’s second studio (section During the week) and before the week ends. “Tutor instructions” were likely to be posted prior to the studio and would consist of reminders of what to submit the following day. Through these categories, we can see how instructors proactively achieved studio goals while sustaining digital engagement with students. Such inference is further supported by claims in Tate and Osborne [60] that increased tutor involvement on SN platforms such as Facebook may enhance engagement. Coupling interpretation of “who” and “when” results suggests that faculty members’ pro-active posts were strategized to align with stages of the design process, as the timing of each post type corresponds with related studio activities.

Post-categories exclusively by students (section “Who?”) also illustrate how the platform encouraged a pro-active, peer-to-peer dialog. For instance, “student-to-student communication” consisted predominantly of original posts that included attachments; mainly CAD files which students shared amongst themselves. “Peer-to-peer learning” usually contained shared posts and were likely to contain images as the predominant attachment-type. Such instances corroborate claims that using SN in higher education may support students’ communicative experiences by leaving messages to be responded to subsequently [7]. Furthermore, these posts were likely to be created on days when the physical studio was not held (section During the semester), indicating that Facebook extended temporal boundaries of the physical design studio [20, 60].

### Re-active engagement

Likes and comments are considered here as the re-active form of engagement on the group, where its users engage reactively to the group’s pro-active form of initial engagement occurring in the form of posts. Descriptive statistics (Figs. 4, 5, 6, and 7) show that most re-active engagement occurs by students. Students liked posts 781 times (i.e., 90% of likes), whereas faculty only liked posts 90 times (10%) during the same period. Out of 53 comments, only 15 were by faculty (28.3%) whereas 38 comments were by students (71.6%). Statistical analyses facilitated identifying distinct patterns of re-active engagement, discussed here.

Faculty was likely to like student-created posts if they were original. Likes by faculty were most likely to occur between weeks 1–3. Likes by students were also significantly associated with the semester’s progression, as these were most likely to occur between weeks 4 and 6. Findings (section During the semester) indicate that the semester’s weekly progression is negatively correlated with likes, which is similar to McCarthy’s [38, 39] findings, also reporting that students were more interactive at the start of the semester, with participation subsequently declining. These engagement patterns may mirror architectural design studio workload distribution, with much of it skewed towards the end of the semester [32].

According to media communications literature [28], the like function tends to be used in response to messages evoking an emotional response, explaining why “tutor-student social interaction” and “fun” posts attained the highest mean rank of likes (section Post-classification). Moreover, high mean rank of likes to “tutor-student social interaction” and “fun” posts corroborate previous literature surrounding student interaction (e.g., [6, 18]) that suggests that students of the Net Generation are more likely to blend between requirements of their schoolwork and socializing online than their elder counterparts and that the boundaries of these two activities are becoming increasingly blurred. Kim and Yang [28] also establish a link between Facebook behaviors and post type, message strategy, and message form. Posts containing images are significantly related to likes but not comments. This is related to the post’s message strategy as the surveyed literature indicated, where users tend to like posts that use visuals that affect emotions or stimulate senses. This may explain the consistently significant relationships in our study between numbers of likes and attachment inclusion in a post, and attachment-type, especially when it includes images or web-links.

Analyses also indicates that images (such as those of design or presentation precedents) are the attachment-type most likely to yield likes, followed by web-links, such as those of online design articles, or digital project galleries (section Attachment). This is comparable to Moore-Russo et al*.* [40], in which images also yielded highest numbers of likes, but disagrees with results in Valerio et al. [62], in which videos were most liked, followed by images and subsequently web-links. Pertinence of attachment-types may be explained using the concept of vividness [17]. Textual posts are least vivid, meaning lower levels of engagement. Images are more vivid than text, while web-links have greater vividness as they may re-direct users toward textual and/or pictorial posts. Videos are considered highly vivid for inclusion of sound. Yet, in our study, posts containing videos received a lower mean rank of likes than posts containing images and web-links. Nevertheless, it is important to consider the “visually-biased” [44] architectural context of this study when interpreting these results.

Users are likely to comment on posts that either contain rational information, soliciting responses or prompting discussion [28], thus explaining why ‘student-tutor enquiries’ and ‘tutor announcements’ categories attained the highest mean rank of comments (section Post-classification) highlighting how both groups communicate. This possibly explains consistently significant relationships between progression of weeks and numbers of comments (section During the semester) as posts consisted of response-soliciting messages related to submission requirements. This also corroborates findings of previous studies where the requirement of tasks, in a context of online learning, impacts the engagement in terms of the type and frequency of cognitive and social activity occurring on the group [43]. Such a re-active behavior may also be explained by the proposed framework of information needs in Chang and Gomes [6]. On the one hand, comments to “student-tutor enquiries” and “tutor announcements” may serve as recognition that information has been attained by students who actively sought it. On the other hand, other students may not have intentionally sought out this same information but upon finding it, may have recognized that it was needed for their course progression, or that it simultaneously addressed some of their unspoken concerns.

The highest mean rank of comments repeatedly occurred prior to an interim submission at week 7, which is comparable to the observation made in Tate and Osborne [60] as most group activity also occurred before a deadline. This result further illustrates how the platform facilitated student engagement corresponding to design process activities.

Nevertheless, juxtaposing significant relationships between all independent variables and numbers of likes and comments (Figs. 6 and 7) shows 19 significant relationships for likes and only 8 for comments. Varying levels of cognitive effort and commitment needed to like and/or comment may explain such discrepancy. Kim and Yang [28] describe liking a post as a “consuming” behavior, i.e., reading content without contributing or creating a new material. Commenting on a post is a “contributing” behavior [28], which is more participative as it requires more effort, time, and commitment.

**Fig. 10:**
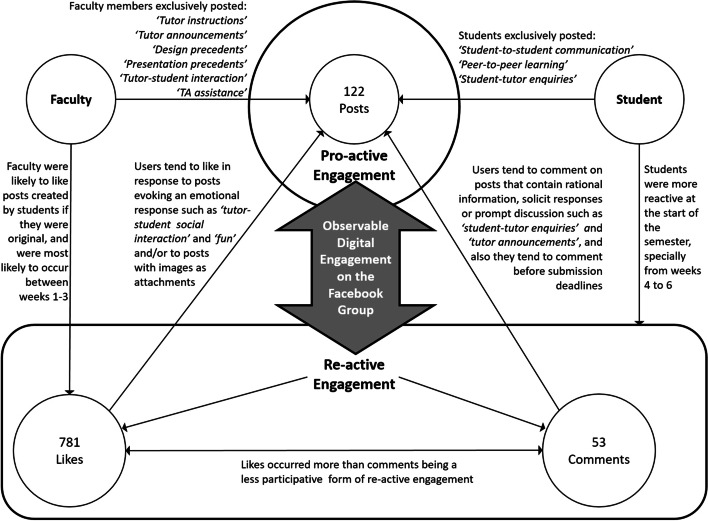
Diagram representing most significant relationships between studied variables

All these discussed significant relationships between the studied variables are presented in Fig. 10 below.

## Conclusions

A quantitative methodology was used to analyze observable online digital engagement on a SN platform that was used as part of a hybrid architectural design studio. The platform’s purpose was to overcome time and distance limitations impinged onto traditional studio set-ups. Using inferential statistics, the study aimed to decipher intricacies regarding how users engage on the platform by answering research questions pertaining to “what,” “who,” and “when” it is used.

Based on the 12-point classification developed by the authors, analysis of the “what” component reveals post-categories under which most posts were created (section Data coding and analysis). Findings suggest that pro-actively creating posts evoking an emotion or soliciting response may encourage engagement. Attachment inclusion is likely to yield likes particularly when the attachment is an image. With respect to “who,” we conclude that increased instructor involvement is associated with greater digital engagement. Findings of the “when” component illustrate how the platform extended the temporal boundaries of the physical studio while aligning with design activities both throughout the week and the semester.

Based on the outcomes, the following techniques are recommended for educators planning online and/or hybrid design studios using SN and seeking to initiate and maintain digital engagement. Tutors’ strategic and continuous active involvement on SN platforms is recommended. Also, creation of content soliciting responses or requiring substantial cognitive effort could ensure engagement throughout different course stages. However, additional social content alongside academic material may encourage further social connections, interactions, and affect emotions, which may also garner further digital engagement and is therefore also recommended. Maintaining the vividness of posts using images or web-links as attachment-types also encourages users to engage.

Additionally, the discussion section of this article, presenting pro-active engagement followed by re-active engagement, may imply that one engagement form always precedes the other. The linear structure of the Facebook platform [29], which requires that posts are pro-actively created before users can respond to re-actively (i.e., liking and commenting), further suggest that engagement mirrors this linear structure. However, Johnston and Lane [27] observe that interaction lies at the core of engagement, and as “interaction is [a] … reciprocal action where two or more parties have an effect upon one another” [19], we can tentatively hypothesize that a cyclical dynamic between both engagement forms on the platform exists, where one informs the other. Re-active engagement may serve as a precursor, indicating to faculty what students need to supplement their teaching/learning or vice versa, stimulating more pro-active engagement reciprocally. However, further research is needed to confirm this conjecture.

The moderately sized sample of 122 posts created by 49 study participants, and the relatively short study period of 16 weeks may initially appear as study limitations, especially when compared to sample sizes in similar research (e.g., over 5000 posts in Cvijikj and Michahelles [12] and over 31,000 posts in Valerio et al., [62]). Nevertheless, the 81% statistical power confirmed from the sample (section Power analysis), outweighs this limitation, as it suggests that all significant results obtained from this sample are noteworthy. However, despite this statistical power, it is plausible that the non-compulsory and non-incentivized environment in which this study was undertaken may have had an impact on the moderate number of posts appearing on the platform during the study period. The sample size raises issues of privacy surrounding SN use and students’ willingness to use their private profiles to voluntarily engage in academic discussions. Such questions therefore point toward a need for a qualitative follow-up of this study, to further understand how specific socio-cultural aspects surrounding the design studio, along with the individual preferences affect the effectiveness of online/hybrid architectural pedagogy.

To the best of our knowledge, this is the first study to comprehensively examine multiple dimensions of SN digital engagement in an architectural design studio, making this a valuable contribution, as schools of architecture worldwide transition to online and/or hybrid design education. Another contribution is the proposition of a 12-point classification that facilitates analyses of social interactions on a platform tailored to architectural contexts and describes the breadth of activity on the platform, which was found to differ in terms of classification of activities from previous studies looking at different fields.

While implications of disease spread on architectural education are still being understood, we uphold that this study holds critical pedagogy potentials. The need to maintain the key value of engagement with students at a distance while preserving the democratic dialogical social component of the design studio is evermore salient as we navigate the COVID-19 reality. Engagement is integral to design education, and this study suggests that it can be maintained digitally using prevalent SN tools, serving as a starting point to plan design education for architectural pedagogy in this post-pandemic narrative.

## Data Availability

The datasets generated during and/or analyzed during the current study are available from the corresponding author on reasonable request for confidentiality reasons.

## Abbreviations

SN: Social networking
VDS: Virtual design studios
LMS: Learning management systems
SNLC: Social network learning cloud

## Footnotes

1 Consistently significant relationships are defined as repeated significances between a single independent variable (e.g., “post-classification,” “shared or not?” “attachment or not?”) and multiple dependent variables (likes and/or comments) at a time, shown in Table 1
